# The Internal Reliability and Construct Validity of the Evidence-Based Practice Questionnaire (EBPQ): Evidence from Healthcare Professionals in the Eastern Mediterranean Region

**DOI:** 10.3390/healthcare11152168

**Published:** 2023-07-31

**Authors:** Naglaa Youssef, Marina Saleeb, Assem Gebreal, Ramy Mohamed Ghazy

**Affiliations:** 1 Department of Medical-Surgical Nursing, College of Nursing, Princess Nourah Bint Abdulrahman University, P.O. Box 84428, Riyadh 11671, Saudi Arabia; 2 Public Health Institute, Faculty of Health, Liverpool John Moores University, Liverpool L2 2QP, UK; 3 Faculty of Medicine, Alexandria University, Alexandria 21568, Egypt; 4 Tropical Health Department, High Institute of Public Health, Alexandria University, Alexandria 21561, Egypt

**Keywords:** Arabic speaking, EBPQ, healthcare professionals, validity

## Abstract

Background: Fostering a culture of clinical effectiveness among healthcare professionals (HCPs) is crucial to achieving optimal patient health outcomes. To our knowledge, there is a lack of robust evidence-based practice (EBP) tools to assess the competence of HCPs in EBP in the Eastern Mediterranean Region (EMR). Aim: This study aims to comprehensively investigate the construct validity and internal reliability of the evidence-based practice questionnaire (EBPQ) among HCPs in the EMR. Methods: This multinational and multi-disciplinary cross-sectional study was conducted between 27 April and 11 May 2023. Convenience and snowball sampling methods were used to recruit a sample of HCPs (physicians, nurses, physiotherapists, dentists, and pharmacists) using an electronic survey questionnaire for data capture. To assess the reliability of the instrument, Cronbach’s alpha, inter-item reliability, and split-half reliability analyses were conducted. Furthermore, the convergent and discriminant validity of the questionnaire was ensured by calculating the average variance extracted (AVE) and the correlation coefficient between the different constructs, respectively. Factor loadings and cross-loadings of different indicators within each construct were calculated by performing both exploratory and confirmatory factor analyses. Results: A total of 1536 HCPs from 18 countries in the EMR (response rate = 96.786%) with a median age of 28 years participated; 47% were female, and 55% had Arabic as their first language. English was the most common language for a bachelor’s degree in science (54%). The construct validity of the EBPQ was investigated using exploratory factor analysis (EFA), which yielded four loaded factors. The confirmatory factor analysis (CFA) confirmed the four loaded factors. The CFA model showed that the root mean square error of approximation = 0.066, comparative fit index = 0.95, Tucker–Lewis’s index = 0.94, standardized root mean square residual = 0.033, normal fit index = 0.94, goodness of fit = 0.91, and χ^2^ test statistic= 22,553, with *p* < 0.001. The AVE values of the four factors were close to 1 (knowledge = 0.6, practice = 0.6, attitude = 0.5, and sharing = 0.7), thus supporting the convergent validity of the EBPQ. The four domains had Cronbach’s alpha coefficients and Omega ≥ 0.7 (knowledge = 0.9, practice = 0.9, attitude = 0.7, and sharing = 0.8), suggesting that the items within each domain had good internal consistency. These results support the discriminant validity of the EBPQ. Conclusions: The EBPQ is a robust questionnaire that can be completed in less than 10 min by EMR HCPs and can be used as a gold-standard questionnaire to collect valid data on the attitudes, knowledge, and proficiency of HCPs in making clinical decisions based on evidence. Future studies are recommended to investigate the retest reliability.

## 1. Introduction

Implementing evidence-based practice (EBP) according to international standards not only ensures the delivery of optimal care to clients [1] but also equips healthcare professionals (HCPs) with a problem-solving approach that enhances the quality of the provided health services [2]. The delivery of consistent and high-quality healthcare services is a major challenge for healthcare systems [3]. There is global recognition of integrating EBP in decision making in health [4,5] and non-health fields [6], which enables individuals to enhance their critical thinking skills, make informed decisions, and practice based on a wide range of trustworthy evidence from multiple reliable sources [6].

EBP initially emerged in medicine as a response to address the practice gap and reduce overreliance on clinical expertise alone in clinical decision making. It aims to shift the focus toward a more reliable and evidence-based approach, where decisions are informed by trustworthy evidence rather than solely relying on individual expertise [6]. In the 1990s, researchers from McMaster University introduced the term EBM, and in 1996, it was defined as a systemic approach to analyze published research as the basis of clinical decision making [7]. Later, Sacket et al. formally defined EBM as ‘the conscientious, explicit, and judicious use of the current best evidence in making decisions about the care of individual patients [8]. Evidence-based medicine (EBM) has been used in various forms such as EBP, evidence-based care (EBC), evidence-based health (EBH), and evidence-based nursing (EBN). However, they share the common premise that patient care should be guided by robust and reliable evidence [9]. The definition of EBP in the nursing profession has evolved from a strict clinical basis to a more holistic approach, based on the full spectrum of nursing research and practice, considering the values of individuals, clinical judgment, ethics and legislation, clinical experience, and practice environments [9]. Therefore, in our study, we used the term EBP to be more applicable to HCPs, regardless of their specialty.

Indeed, fostering a culture of clinical effectiveness among HCPs is crucial to achieve optimal patient health outcomes. Recognizing the significance of EBP, it has been declared an essential element of the health education curriculum [10,11]. Research has shown that a well-developed EBP course can improve undergraduate attitudes toward applying EBP after graduation. The EBP course equips undergraduate students with the necessary knowledge, skills, and confidence to engage in evidence-based decision making in their future practice [12].

Despite the potential benefits of EBP, its application remains limited in many healthcare settings especially in the Eastern Mediterranean Region (EMR) [13]. The EMR faces numerous challenges, including critical deficits in human resource development, resources, and research training. These limitations hinder the translation of the research into policy and practice. One of the key factors contributing to this issue is the weakness of institutional and financial inducements within the research and development systems [13].

In the rapidly evolving healthcare field, it is vital to regularly assess the attitudes, skills, and knowledge of HCPs regarding EBP. This ongoing evaluation helps us to identify obstacles and challenges that enable appropriate actions to be taken. Moreover, determining HCPs’ readiness and willingness to implement EBP is crucial for sustaining a culture of EBP [14]. Given the lack of evidence concerning the competence of HCPs in the EMR regarding EBP and the perceived barriers to its adoption in clinical decision making, it is essential to conduct a large survey among HCPs in the EMR. Our study is part of a multinational project in the EMR, which aims to identify the barriers facing EBP implementation in the region. Therefore, it was essential to search and find a well-constructed and reliable scale that can achieve our study objectives.

Several EBP scales have been developed, including the Health Sciences Evidence-Based Practice questionnaire (HS-EBP) (60 items) [15], Evidence-based Practice Competence Questionnaire (EBP-COQ) (25 items) [16], McEvoy trans-professional instrument of evidence-based practice profile (EBP2) (49 items) questionnaire [17], Competencies, Beliefs, Facilitators, Barriers, Implementation of Evidence-Based Practice (EBP-CBFRI) (55 items) [18], and Evidence-Based Practice Questionnaire (EBPQ) (24 items) [19]. However, while some are too long [15,17,18], available in Spanish [15,16], and for nursing students [16], the EBPQ is a concise and comprehensive tool in English [19]. Moreover, the EBPQ contains all the EBP domains, which makes it a widely used tool among HCPs [20,21,22]. However, in the EMR, the validity and reliability of the EBPQ among HCPs have not been reported yet until the current study, which could provide essential new information about the attitude and competence of HCPs in the EMR regarding EBP.

Since most EMR countries speak Arabic as their first language and study the degree of health sciences in English, it was appropriate to administer the EBPQ in its original language (English) to investigate its validity and reliability. Additionally, the EBP is a model that is used globally under the same themes, terms, and processes, and it is taught and administered in English among HCPs in the majority of EMR countries. Therefore, it is logical to administer the EBPQ in its original language without translation in order to assess its validity and reliability before use in the main project.

### Aim

This study thoroughly examined the construct validity and internal reliability of the EBPQ (English version) among EMR HCPs.

## 2. Construct Validity and Internal Reliability of the EBPQ

This study was conducted in the following three phases.

### 2.1. Phase-1: Ensuring Face and Content Validity of the EBPQ

A panel of experts of eight HCPs, who were specialists in medicine, nursing, and pharmacology, was invited to check and provide feedback on the content, readability, and understandability of the EBPQ (English version). As shown in Table A1, the panel committee was selected from three of the EMR regions, and their first language is not English, but they studied for their undergraduate degree in English as participants from Egypt and Pakistan, and one from a country where the health sciences are taught in Arabic, but they use English as a second language, like a panel member from Syria. The panel reported that the questionnaire items were clear, simple, and understandable and that they were familiar with them. As a result, there was no change in the EBPQ’s words or sentence structure, and they approved of its face and content validity.

### 2.2. Phase-2: Conducting the Cognitive Debriefing by Pilot Sample

We sent an electronic version of the English EBPQ to 26 HCPs from 13 countries (Afghanistan, Djibouti, Egypt, Jordan, Lebanon, Libya, Morocco, Pakistan, Saudi Arabia, Somalia, Syrian Arab Republic, Tunisia, and Yemen) via WhatsApp, requesting them to complete the questionnaire and report its clarity, readability, understandability, and the time taken to complete it. All participants completed the questionnaire within 3–6 min. All participants considered the questionnaire to be easy to read, clear, and relevant. None of the participants suggested any modification.

### 2.3. Phase-3: Assessment of the Validity and Reliability of the EBPQ

Construct validity and internal reliability of the EBPQ were investigated using a cross-sectional survey. The ‘Strengthening the Reporting of Observational Studies in Epidemiology (STROBE)*’* was followed while conducting this study [23].

## 3. Materials and Methods

### 3.1. Design and Setting

A multinational cross-sectional study was conducted from 27 April to 11 May 2023 to recruit a representative and suitable sample from the EMR countries to investigate the construct validity and internal reliability of the EBPQ.

### 3.2. Population and Sampling Method

Convenience and snowball sampling methods were used to recruit a sample of HCPs (physicians, nurses, physiotherapists, dentists, and pharmacists) who met the following eligibility criteria for participation: age ≥ 18 years, HCPs (in the internship year or post-graduation), residence/work in an EMR country during the data collection period, and agreed to fill out the electronic questionnaire.

### 3.3. Sample Size

The sample size calculation depended on the number of questions included in the validation (24 items) and the estimated Cronbach’s alpha, with a 95% confidence interval. The equation suggested by Bonett (2002) [24] was used to calculate the sample size: n = {8k/(k − 1)}{Z critical/ln((1 − pk − e/2)/(1 − pk + e/2))}^2 + 2, where n is the sample size, k is the number of the questions used in the validation, Z critical is the critical value of the Z score at a significance level of 5%, pk is the estimated Cronbach’s alpha level, and e is the absolute difference between the upper and lower bounds of the confidence interval of the estimated alpha level. It was estimated that the alpha level is 0.7 (acceptable level), the absolute difference in CI (e) is 0.2, and after substitution in the equation, the sample size would be n = (8 × 24/23) ((1.96/ln (2))^2 + 2 = 8.4 × 8 + 2 = 69 participants per each of the EMR countries. In total, 1587 HCPs from 18 countries in the EMR accessed the questionnaire, and 51 refused to participate, ending with 1536 actual participants (a response rate of 96.786%). Our sample was larger than the computed size, which could also be used to run a confirmatory factor analysis test.

### 3.4. Instruments

The electronic survey used to collect data included two questionnaires.

#### 3.4.1. Sheet of Background Data about the Participants

This part of the questionnaire consisted of nine questions about the demographic and academic characteristics of the participants and training in EBP (i.e., age, gender, country of residence, first language, highest education degree, field of practice, language of the Bachelor of Science, current work status, and type of hospital where their current training or work was conducted). In addition, four questions were used to collect data about HCP training in EBP, as recommended by [16]: (1) studying the EBP course in college before graduation, (2) attending training in EBP, (3) number of studies carried out in the last three years, and (4) number of research articles that they read in the last month. For participants with a native language other than English, an additional item was added to evaluate whether their ability in English is a barrier to using research-based evidence, as recommended by [25].

#### 3.4.2. The Evidence-Based Practice Questionnaire (EBPQ)

The EBPQ measures knowledge, skills, and attitudes toward EBP. It includes 24 items comprising three subscales: (i) self-reported practice or use of EBP (practice subscale) (6 items), (ii) EBP attitudes (attitude subscale) (4 items), and (iii) EBP knowledge and skills (knowledge subscale) (14 items) [19]. All items were rated on a Likert scale ranging from 1 to 7. The instrument score was calculated by adding the response values to each question, giving a total of 168 points, with higher scores indicating more positive attitudes toward EBP [26]. The practice (42 points), attitude (28 points), and knowledge (98 points) subscales were used in this study. The instrument had a Cronbach’s alpha of 0.87 and satisfactory convergent validity (*p* < 0.001) [19]. A previous study showed that the Cronbach’s alpha of different sections of the questionnaire ranged from 0.7 to 0.9 among academic nursing educators in Egypt and Jordan [27].

### 3.5. Recruitment Method

An anonymous electronic self-administered survey using the ‘Google Form’ was generated. The link to the electronic survey was then sent to colleagues in the health field of each of the targeted countries. They were invited to complete the questionnaire and send it to their colleagues via WhatsApp and other social media platforms. The participants were able to fill out the questionnaire only one time.

### 3.6. Ethical Considerations

The Institutional Review Board (IRB) exempted the study (IRB Log Number: 23-0421) as it posed minimal risk to the participants. The purpose of the study was presented on the survey cover page. Anonymity was ensured by not collecting identifiable personal data, such as names and workplace names, and by ensuring that their data were confidentially protected and that the collected data would only be accessed by the study researcher. The confidentiality of the participants was also ensured by only using the coded responses while analyzing the results of the survey. Participants were informed that they had the right to withdraw from the study without any consequences and that their participation was voluntary. The collected data were protected by saving them on a secured laptop which was only available to the researchers.

### 3.7. Data Analysis

Statistical analyses were performed using IBM SPSS version 26 (IBM SPSS, Armonk, NY, USA). Descriptive statistics were used to determine the frequency, prevalence, percentage, mean, and standard deviation. The Shapiro–Wilk test was used to check the normality of continuous data. As all continuous data did not follow parametric assumptions, they were described using medians and interquartile ranges. All categorical variables were described as counts and percentages. An independent *t*-test was performed to compare the EBP domains between groups who experienced difficulties in the English language and those who did not, as well as between those who took EBP courses at the university and those who did not. The internal consistency of the questionnaire was assessed by calculating the Cronbach’s alpha, inter-item correlations, and split-half reliability. The acceptable alpha levels start from 0.7, and when the levels reach closer to 1, the internal consistency is better explained [28]. To assess convergent validity, the average variance extracted (AVE), which is the average variance in indicator variables explained by a construct, was calculated. If the AVE is greater than or equal to 0.5, convergent validity is confirmed [29]. Discriminant validity was assessed by calculating the correlation coefficients between the different constructs. Discriminant validity was ensured when the correlations were not statistically significant. Factor loadings and cross-loadings were produced using exploratory factor analysis (EFA). Confirmatory factor analysis (CFA) was performed to verify the overall factor structure and calculate the significance level of the loadings. R software (version 4.1.1) was used to calculate the model fit of the CFA that was used to assess the construct validity of the questionnaire. Statistical significance was set at *p* < 0.05.

## 4. Results

### 4.1. Characteristics of HCPs

A total of 1536 HCPs participated in this study, with a median age of 28 years (range 25–32 years). Of the total participants, 47% were female, and nearly half (55%) had Arabic as their first language. The distribution of education degrees shows that 9.4% had a doctoral degree, 27% had a master’s degree, 47% had a bachelor’s degree, and 16% were still in their internship years. The participants had diverse fields of practice, with dentistry (18%), medicine (36%), nursing (21%), pharmacy (13%), and health and rehabilitation sciences (12%) being the main areas. English was the most common language for a bachelor’s degree in science (54%), followed by Arabic (17%).

Nearly two-thirds (58%) of the participants worked full-time, 29% worked part-time, and 12% were not currently working (on vacation, retired, or volunteering). The median number of work experiences reported by the participants was four (2–7) years. Participants worked or were trained in various types of hospitals, including public/teaching/government hospitals (39%), private hospitals (23%), private and public hospitals (22%), and others (16%) (Table 1).

### 4.2. Experience of HCPs in Research and EBP

A total of 52% had studied EBP in college before graduation, whereas 55% had attended EBP training. The median number of research studies conducted in the last three years was 1.0 (0.0–3.0). Additionally, participants read a median of two research articles in the last month (0–5). A total of 52.6% (n = 808) reported that the research articles in English were not difficult to read, whereas the rest (n = 728, 47.4%) reported that it was difficult (Table 1).

### 4.3. Reliability and Divergent Validity of the EBPQ

Descriptive statistics, Cronbach’s alpha, item-total score correlation, and split-half reliability for each domain are presented in Table 2.

The practice domain consisted of six items (P1–P5). The raw alpha coefficient for each item in the practice domain was 0.8, indicating good internal consistency. The item-total domain correlation ranged from 0.6 to 0.7, suggesting that the items were moderately correlated with the overall domain (convergent validity). The overall Cronbach’s alpha for the practice domain was 0.86 (with a 95% CI: 0.85–0.87), indicating high internal consistency. The split-half reliability was 0.8, indicating a high level of internal consistency between the two halves of the questionnaire. The Spearman–Brown correction was 0.9, suggesting that if the questionnaire length increased or decreased, the reliability of the measure would remain relatively stable (Table 2).

The attitude domain included three items (A1–A3). The raw alpha coefficient for each item of the attitude domain ranged from 0.6 to 0.7, indicating acceptable internal consistency. The item-total domain correlation ranged from 0.5 to 0.6, indicating moderate correlations with the overall domain (convergent validity). The overall Cronbach’s alpha for the attitude domain was 0.74 (with a 95% confidence interval of 0.72–0.76), suggesting good internal consistency. The split-half reliability was 0.6, and the Spearman–Brown correction was 0.7 (Table 2).

The knowledge domain comprised 13 items (K1–K13). The raw alpha coefficient for each item in the knowledge domain was 0.9, indicating excellent internal consistency. The item-total domain correlation ranged from 0.7 to 0.8, indicating strong correlations with the overall domain (convergent validity). The overall Cronbach’s alpha for the knowledge domain was 0.95 (with a 95% confidence interval of 0.94–0.95), indicating high internal consistency. The split-half reliability was 0.9, and the Spearman–Brown correction was 0.9 (Table 2).

Three items were removed from the EBPQ, which had a high uniqueness value (r > 0.6): (1) item 6 of the practice ‘shared this information with colleagues’, (2) item 1 of the attitude ‘My workload is too great for me to keep up to date with all the new evidence’, and (3) item 14 of knowledge ‘Ability to review your own practice’.

### 4.4. Construct Validity of the EBPQ

#### 4.4.1. Exploratory Factor Analysis

The ‘Kaiser–Meyer–Olkin factor adequacy and Bartlett test’ showed that the overall measure of sampling adequacy (MSA) was 0.97, suggesting that the data used in the factor analysis were highly suitable for conducting the analysis. The MSA values were closer to 1, indicating that the variables in the analysis were highly correlated and provided a good basis for factor extraction. The Bartlett test was <0.05, indicating rejection of the null hypothesis, as there was a significant correlation among the variables. Factor analysis revealed that four factors (Factor 1, Factor 2, Factor 3, and Factor 4) were extracted. Factor 1 had relatively high loadings for items K1–K11. Factor 2 had relatively high loadings for items P1–P4. Factor 3 showed high loadings for items A1–A3. Factor 4 had high loadings for items K12–K13. The uniqueness values of the items ranged from 0.3 to 0.6, indicating that each item had a moderate-to-high level of uniqueness (Table 3).

#### 4.4.2. Confirmatory Factor Analysis (CFA)

**Figure 1:**
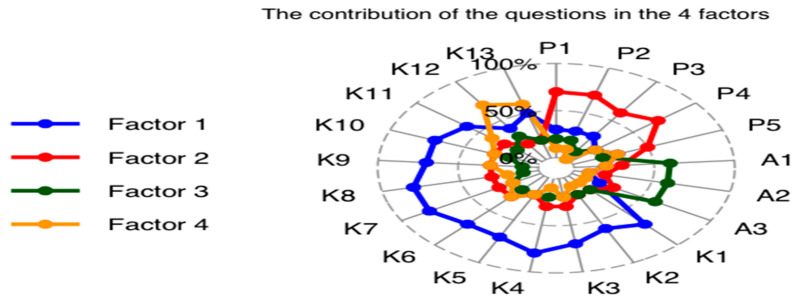
Radar plot to show the factor loadings.

**Figure 2:**
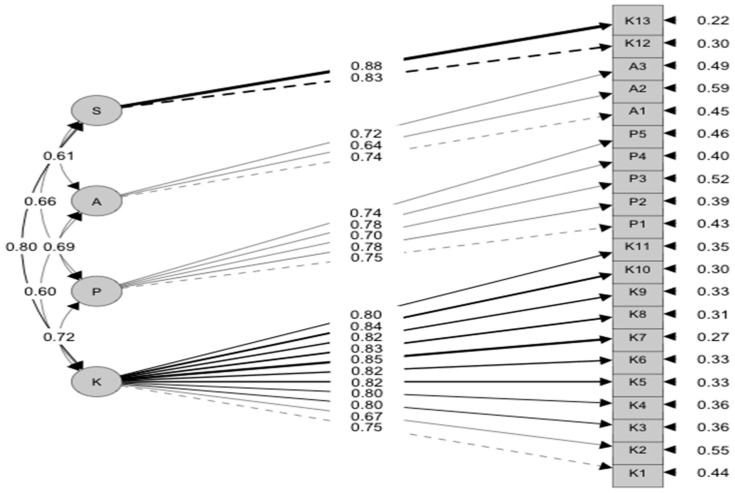
Confirmatory factor analysis.

Construct validity and model fit measures were investigated using confirmatory factor analysis (CFA). The fit of the model was evaluated using several indicators: root mean square error of approximation (RMSEA = 0.066), comparative fit index (CFI = 0.95), Tucker–Lewis index (TLI = 0.94), standardized root mean square residual (SRMR = 0.033), normal fit index (NFI = 0.94), goodness of fit (GFI = 0.91), and χ^2^ test statistic = 22,553 with 210 degrees of freedom and *p* < 0.001 (Figure 1 and Figure 2).

#### 4.4.3. Convergent Validity of EBPQ

The AVE is a measure of convergent validity that represents the average amount of variance captured by the items within each variable. The AVE values ranged from 0 to 1, with higher values indicating stronger convergent validity (knowledge = 0.6, practice = 0.6, attitude = 0.5, and sharing = 0.7). These values suggest that the items within each variable capture moderate-to-high variance, which supports convergent validity.

### 4.5. The Internal Reliability

The four domains had Cronbach’s alpha coefficients and Omega ≥ 0.7 (knowledge = 0.9, practice = 0.9, attitude = 0.7, and sharing = 0.8), suggesting that the items within each domain had good internal consistency. Omega is another measure of internal consistency reliability that showed values for the four domains similar to the alpha values, which indicates that the EBPQ has a strong internal consistency.

### 4.6. Divergent (Discriminant) Validity

The correlation between the four domains using the Pearson method showed that the correction between knowledge and practice was −0.5, and between knowledge and attitude, it was −0.6, indicating a moderately negative relationship. The correlation between practice and attitude was −0.2, indicating a weak negative relationship. The correlations between sharing and the other three domains (knowledge, practice, and attitude) were relatively weak and non-significant (0.1, −0.4, −0.1, respectively). These findings indicate that the EBPQ has good divergent validity.

### 4.7. Known Group Validity

Known group validity is the ability of a questionnaire to discriminate between groups that are known to have significant differences. We hypothesized that the mean scores on the EBPQ subscales would be significantly lower among participants who had fewer years of work experience, had experienced difficulty in English research articles, read fewer research articles, and did not receive EBP training before graduation. The results in Table 4 and Table 5 support the discriminant validity of the EBPQ.

## 5. Discussion

Our study is the first to investigate the construct validity and internal reliability of the EBPQ among HCPs in the EMR. The original EBPQ was developed in English to assess United Kingdom (UK) nurses’ knowledge, skills, and attitudes toward EBP [19]. The EBPQ is a short questionnaire that is widely used to evaluate attitudes, knowledge, and skills of EBP [20,21,22,27]. This makes it an appropriate tool that can be used as a standardized questionnaire to compare the attitudes, knowledge, skills, and practice of EBP by HCPs in EMR countries because the majority of HCPs in the EMR use English as a secondary language in health sciences colleges and clinical practice. Thus, the original version of the EBPQ was applied without translation into Arabic. We assume that HCPs should be well-prepared to adopt EBP in clinical decision making and that they must critically understand the scientific research and guidelines that are usually available in English.

### 5.1. Construct Validity

First, the 24 items of the EBPQ were tested for uniqueness using a correlation test. Three items were removed from the EBPQ, as they had a high correlational value that decreased the construct validity of the tool. When the 21 items were entered into the EFA, four factors were extracted, and the CFA proved that Factor 1: Knowledge, Factor 2: Practice, Factor 3: Attitude, and Factor 4: Sharing. The fourth newly extracted domain had two items that were loaded under knowledge in the original EBPQ: (1) item 12 ‘Share of ideas and information with colleagues’ and (2) item 13 ‘Dissemination of new ideas about care to colleagues’. This finding makes sense since these two items assess the same construct related to the sharing and dissemination of research ideas and are not related to the ‘knowledge domain’ as in the original EBPQ. Therefore, the fourth domain was labeled as ‘sharing EBP’. This finding proved the internal stability and construct validity of the EBPQ among the study participants. Therefore, the final version of our EBPQ had 21 items that could be integrated into four domains.

### 5.2. Divergent Validity of the EBPQ

Our findings indicated that the EBPQ had good discriminant validity, as it could differ between groups that were hypothesized to have different attitudes, knowledge, skills, and sharing. For example, our findings showed that HCPs who experienced difficulty with English research articles had lower EBPQ scores than those who reported no difficulty. Furthermore, the mean score for sharing research ideas (domain 4) significantly increased with increasing age, work experience, previous study of EBP courses, the number of research articles they published, and the number of research articles they read. In addition, scores for the attitude domain significantly increased with increasing age and work experience. Interestingly, scores for the practice domain increased significantly with an increase in reading research articles and previous study of EBP. This finding is important because it reflects the necessity of reading more research articles which, at the same time, requires high English proficiency and the importance of impeding EBP courses in the undergraduate curriculum. Attitude varies significantly with age and experience but not with the number of research studies carried out or the number of research articles read. This finding supports that our study participants have a good attitude toward EBP regardless of whether they have research experience; however, this experience seems necessary to improve their attitude toward EBP. In the United Arab Emirates, older nurses with more years of experience and higher education levels had significantly higher mean EBP scores. In addition, gender is significantly linked to practice and knowledge [30].

The critical factor significantly associated with practice, skill, and sharing was the number of research articles read. A recent systematic review concluded that research training is an important factor in practice-based research among pharmacists [31]. In Qatar, post-graduate qualifications were associated with readiness to practice research after adjusting for gender and time since the completion of the undergraduate study [32]. These findings support the importance of practicing research through reading and engaging in research activities to enhance practice-based research.

### 5.3. Internal Reliability

Descriptive statistics, Cronbach’s alpha, item-total score correlation, and split-half reliability were calculated for each domain. Cronbach’s alpha showed that the EBPQ reliability for the attitude, practice, and knowledge domains (ranging from 0.74 to 0.95, respectively) exceeded the set criteria, indicating its high internal consistency, which was almost higher or similar to that of a previous study [19,33,34].

However, attitude had the lowest Cronbach’s value, similar to the original study by Upton and Upton [19], even in a study that translated and tested the same tool among Indonesians, where its overall Cronbach’s α was 0.92 (with α of practice = 0.81, attitude = 0.81, and knowledge = 0.94) [21].

## 6. Conclusions

Our study showed that the EBPQ may be a useful scale that can be used among HCPs in the EMR, especially those who study health sciences in English. The EBPQ is a robust and self-administered questionnaire that can be completed in 10 min by EMR HCPs. Therefore, the EBPQ can be used by health researchers and policymakers as a gold-standard questionnaire to collect valid data on the attitudes, knowledge, and proficiency of HCPs in making clinical decisions based on evidence to improve the quality of healthcare. Moreover, it can be used to compare EMR countries and evaluate the effectiveness of EBP training programs.

## 7. Limitations and Recommendations

Our study is the first to evaluate the validity and reliability of the English EBPQ among a large sample of HCPs from five general health fields in EMR countries, which makes our study a valuable contribution to the existing literature. However, several limitations of this study should be considered in the future. First, since our study recruited the sample using an anonymous electronic survey, participants who could understand English were more willing to fill out the questionnaire than those who could not. Therefore, the English version of the EBPQ is unsuitable for HCPs who do not speak English. Therefore, future studies are needed to translate and adapt the EBPQ to EMR HCPs, who have limitations in the English language. Second, although we recruited a sample larger than the calculated size from 18 countries, using a nonrandom method may limit the generalizability of the study findings to all HCPs in the EMR. Third, investigating the retest reliability was impossible because collecting personal information, such as email, was prohibited to maintain the confidentiality of the participants. Therefore, a future study is recommended to investigate retest reliability, as this was not examined in the present study.

## Figures and Tables

**Table 1:** Sociodemographic characteristics (N = 1536).

Variables		N (%)
Age	Median (IQR)	28 (25, 32) *
Gender	Female	721 (47%)
	Male	815 (53%)
First language	Arabic	851 (55%)
	English	36 (2.3%)
	French	5 (0.3%)
	Other	644 (42%)
The highest education degree	Doctorate degree	144 (9.4%)
	Master degree	422 (27%)
	Bachelor degree in science	723 (47%)
	Still in the internship year	247 (16%)
Field of practice	Dentistry	277 (18%)
	Health and rehabilitation sciences	183 (12%)
	Medicine	555 (36%)
	Nursing	319 (21%)
	Pharmacy	202 (13%)
Language of the Bachelor of Science	Arabic	258 (17%)
	English	837 (54%)
	French	137 (8.9%)
	Other	304 (20%)
Current work status	Not working (in vacation, retired, volunteers)	192 (12%)
	Working full-time	896 (58%)
	Working part-time	448 (29%)
	Number of work experience	4 (2, 7) *
Type of hospital where you currently train or work	Private and public	338 (22%)
	Private hospital	351 (23%)
	Public/teaching hospital/government hospital	605 (39%)
	other	242 (16%)
Studied an evidence-based practice course in college before graduation	No	735 (48%)
	Yes	801 (52%)
Attended training in evidence-based practice	No	689 (45%)
	Yes	847 (55%)
Number of research carried out in the last three years	Number of research you carried out in the last three years	1.0 (0.0, 3.0) *
Number of research articles you read in the last month	Number of research articles you read in the last month	2 (0.0, 5.0) *
Difficulty to understand English research articles Mean = 3.47 ± (SD) 1.301 Median = 4.0	Strongly agree	130 (8.5%)
	Agree	268 (17.4%)
	Neither agree/disagree	330 (21.5%)
	Disagree	359 (23.4%)
	Strongly disagree	449 (29.2%)

Median (IQR) *.

**Table 2:** Reliability and divergent validity of the Evidence-Based Practice Questionnaire (EBPQ) and its three domains.

Items	Raw Alpha	Item-Total Domain Correlation	Mean ± SD	Overall Cronbach’s Alpha with 95% CI for Each Domain	Split-Half Reliability and Spearman–Brown Correction
**1-Practice (P)**					
P1	0.8	0.7	4.0 ± 1.7	0.86 (0.85–0.87)	Split half = 0.8 SB correction = 0.9
P2	0.8	0.7	4.0 ± 1.7		
P3	0.8	0.6	3.8 ± 1.6		
P4	0.8	0.7	4.3 ± 1.8		
P5	0.8	0.7	4.5 ± 1.8		
**2-Attitude (A)**					
A1	0.7	0.6	4.5 ± 1.8	0.74 (0.72–0.76)	Split half = 0.6 SB correction = 0.7
A2	0.7	0.5	4.5 ± 2.1		
A3	0.6	0.6	4.4 ± 1.8		
**3-Knowledge (K)**					
K1	0.9	0.7	4.1 ± 1.8	0.95 (0.95–0.96)	Split half = 0.9 SB correction = 0.9
K2	0.9	0.7	4.1 ± 1.7		
K3	0.9	0.8	4.3 ± 1.7		
K4	0.9	0.8	4.1 ± 1.8		
K5	0.9	0.8	4.3 ± 1.7		
K6	0.9	0.8	4.5 ± 1.7		
K7	0.9	0.8	4.3 ± 1.7		
K8	0.9	0.8	4.2 ± 1.7		
K9	0.9	0.8	4.3 ± 1.7		
K10	0.9	0.8	4.4 ± 1.7		
K11	0.9	0.8	4.5 ± 1.7		
K12	0.9	0.7	4.8 ± 1.8		
K13	0.9	0.7	4.6 ± 1.8		

Overall Cronbach’s alpha (95% CI) = 0.95 (0.94–0.95). Split-half reliability for all the questionnaires = 0.94. Spearman–Brown correction = 0.97.

**Table 3:** Factor loadings and uniqueness of the EBPQ items.

Items	Factor 1 (Knowledge)	Factor 2 (Practice)	Factor 3 (Attitude)	Factor 4 (Sharing)	Uniqueness Value
P1	0.3	0.7	0.2	0.1	0.4
P2	0.3	0.7	0.2	0.1	0.4
P3	0.3	0.6	0.1	0.1	0.5
P4	0.2	0.7	0.2	0.2	0.4
P5	0.3	0.5	0.2	0.3	0.5
A1	0.2	0.3	0.6	0.2	0.5
A2	0.1	0.2	0.6	0.1	0.5
A3	0.2	0.3	0.6	0.1	0.5
K1	0.7	0.2	0.2	0.1	0.4
K2	0.6	0.2	0.2	0.1	0.5
K3	0.7	0.3	0.2	0.2	0.4
K4	0.8	0.3	0.2	0.1	0.3
K5	0.7	0.2	0.2	0.2	0.3
K6	0.7	0.3	0.2	0.3	0.3
K7	0.8	0.3	0.2	0.2	0.3
K8	0.8	0.3	0.1	0.2	0.3
K9	0.7	0.3	0.1	0.3	0.3
K10	0.7	0.3	0.2	0.3	0.3
K11	0.6	0.3	0.2	0.4	0.3
K12	0.4	0.2	0.3	0.7	0.3
K13	0.5	0.2	0.2	0.6	0.3

Highlights showes the factor loading.

**Table 4:** Comparison of EBP between groups who experienced difficulty in English and those who studied the EBP course in college.

EBPQ	English Difficulty	No	Mean	SD	t *	Sig. (2-Tailed)	95% Confidence Interval of the Difference	
							Lower	Upper
Practice	No	808	22.91	6.06	13.86	<0.01	3.96	5.26
	Yes	728	18.29	6.98				
Attitude	No	808	15.10	4.30	15.62	<0.01	3.00	3.87
	Yes	728	11.66	4.31				
Skill	No	808	51.79	13.13	13.43	<0.01	8.61	11.56
	Yes	728	41.70	16.26				
Sharing	No	808	10.43	2.84	13.53	<0.01	1.83	2.45
	Yes	728	8.28	3.36				
**EBPQ**	**Studied the EBP Course in College**	**No**	**Mean**	**SD**	t *	**Sig. (2-Tailed)**	**95% Confidence Interval of the Difference**	
							**Lower**	**Upper**
Practice	No	735	19.38	7.36	−7.40	<0.01	−3.25	−1.88
	Yes	801	21.95	6.21				
Attitude	No	735	12.93	4.75	−4.36	<0.01	−1.48	−0.56
	Yes	801	13.96	4.47				
Skill	No	735	43.17	16.38	−9.53	<0.01	−8.86	−5.84
	Yes	801	50.53	13.82				
Sharing	No	735	8.87	3.43	−6.23	<0.01	−1.35	−0.71
	Yes	801	9.90	3.05				

* Independent-sample test with equal variances assumed.

**Table 5:** Correlation between the EBPQ domains and other variables.

EBPQ—For Domains	r/*p*-Value *	Age	Experience	Number of Research Carried out	Number of Read Research Articles
Practice	r	0.036	0.047	0.036	0.110
	*p*-value	0.157	0.065	0.154	<0.0001
Attitude	r	0.137	0.107	0.018	0.040
	*p*-value	0.000	0.000	0.470	0.115
Skill	r	0.010	0.055	0.048	0.114
	*p*-value	0.706	0.031	0.062	<0.0001
Sharing	r	0.088	0.120	0.066	0.095
	*p*-value	0.001	0.000	0.010	<0.0001

* Correlation at 2-tailed with controlling the variable of ‘feeling difficult of English’.

## Data Availability

Data can be obtained from the corresponding author by email.

## Appendix A

**Table A1:** Characteristics of the panel committee.

Country	Specialty (No)	First Language	Second Language	Undergraduate Study Language
Egypt	Medicines (2)	Arabic	English	English
	Nursing (3)			
	Pharmacy (1)			
Syria	Medicine (1)	Arabic	English	Arabic
Pakistan	Medicine (1)	Ordo	English	English
